# Retraction: Boric acid in magnetized water: clean and powerful media for synthesis of 3,4-dihydropyrimidin-2(1*H*)-ones

**DOI:** 10.1039/d1ra90141a

**Published:** 2021-09-08

**Authors:** Vahid Khakyzadeh, Ahmad Reza Moosavi-Zare, Sahra Sheikhaleslami, Amir Ehsani, Salbin Sediqi, Mohammad Rezaei-Gohar, Zahra Jalilian

**Affiliations:** Department of Chemistry, K. N. Toosi University of Technology P.O. Box 16315-1618 15418 Tehran Iran v.khakyzadeh@kntu.ac.ir; Hamedan University of Technology, Department of Chemistry Hamedan 65155 Iran; Chemistry Department, College of Science, University of Kurdistan Pasdaran Street Sanandaj 66177-15177 Iran

## Abstract

Retraction of ‘Boric acid in magnetized water: clean and powerful media for synthesis of 3,4-dihydropyrimidin-2(1*H*)-ones’ by Vahid Khakyzadeh *et al.*, *RSC Adv.*, 2021, **11**, 22751–22755. DOI: 10.1039/D1RA03769B

We, the named authors, hereby wholly retract this *RSC Advances* article. Although we maintain that the results obtained in distilled water are accurate, and believe that further experiments will confirm our conclusions, following discussions between the authors and the Royal Society of Chemistry, we have determined that the evidence presented regarding magnetised water in this paper is insufficient to support the conclusions and needs further investigation. We are therefore retracting the paper to maintain the validity of the scientific record. The Royal Society of Chemistry apologises for the fact that these concerns were not identified during the peer review process.

Signed: Vahid Khakyzadeh*^,a^, Ahmad Reza Moosavi-Zare^b^, Sahra Sheikhaleslami^a^, Amir Ehsani^a^, Salbin Sediqi^a^, Mohammad Rezaei-Gohar^b^ and Zahra Jalilian^c^

Date: 27^th^ August 2021.

Retraction endorsed by Laura Fisher, Executive Editor, *RSC Advances*, 27^th^, August 2021.

## Supplementary Material

